# Stage-dependent association of BDNF and TGF-β_1_ with lung function in stable COPD

**DOI:** 10.1186/1465-9921-13-116

**Published:** 2012-12-17

**Authors:** Paul Stoll, Urs Wuertemberger, Kai Bratke, Christiana Zingler, J Christian Virchow, Marek Lommatzsch

**Affiliations:** 1 Department of Pneumology and Critical Care Medicine, University of Rostock, Ernst Heydemann Strasse 6, 18057, Rostock, Germany; 2 Institute for Laboratory Medicine, University of Rostock, Ernst Heydemann Strasse 6, Rostock, 18057, Germany

**Keywords:** COPD, Inflammation, Fibrosis, BDNF, TGF-β_1_

## Abstract

**Background:**

Chronic Obstructive Pulmonary Disease (COPD) is characterised by complex inflammatory, neuronal and fibrotic changes. Brain-derived Neurotrophic Factor (BDNF) is a key regulator of neuronal plasticity, whereas Transforming Growth Factor-β_1_ (TGF-β_1_) plays a crucial role in tissue repair and emphysema pathogenesis. Both mediators are stored in platelets and released from platelets in inflammatory conditions and during serum preparation. In patients with asthma, it was previously shown that elevated serum BDNF concentrations correlate with disease severity, whereas TGF-β_1_ concentrations were normal.

**Methods:**

In the present study, 63 patients with stable COPD (spirometric GOLD stages 2–4) and 17 age- and comorbidity-matched controls were studied. Lung function, smoking history, medication, platelet concentrations in peripheral blood and serum concentrations of BDNF, TGF-β_1_ and Serotonin (5-HT) were assessed in all participants.

**Results:**

Serum levels of both BDNF and TGF-β_1_ (but not concentrations of platelets in peripheral blood) were significantly elevated in all stages of COPD as compared to controls. Highest BDNF concentrations were found in spirometric GOLD stage 3, whereas highest TGF-β_1_ serum levels were found in spirometric GOLD stage 4. There were specific, stage-dependent correlations of these mediators with lung function parameters of the patients.

**Conclusions:**

Taken together, we show that, in contrast to asthma, COPD is characterised by elevated concentrations of both BDNF and TGF-β_1_ in serum. The stage-dependent association with lung function supports the hypothesis that these platelet mediators may play a role in the pathogenesis of COPD.

## Background

Chronic obstructive pulmonary disease (COPD), a major cause of morbidity and mortality worldwide [1], is characterised by airflow limitation, predominantly in the small airways, and the development of emphysema [2]. The underlying abnormality is an ill defined, chronic inflammatory process leading to remodeling of the airway and alveolar walls [2,3]. In addition, systemic changes play an important role in the pathogenesis of the disease [4]. The complex fibrotic and neuromuscular changes in COPD are in part different from those changes observed in patients with asthma [5]. Current pharmacotherapy of COPD has only limited impact on airway and alveolar remodeling. Therefore, it is essential to better understand the mechanisms of tissue remodeling in this devastating disease [5].

A pilot study examining serum concentrations of 92 inflammation-associated analytes suggested that Brain–derived neurotrophic factor (BDNF) is among the three most highly elevated mediators in COPD [6]. Furthermore, a microarray analysis of 143 serum analytes postulated that BDNF is the strongest predictor of reduced FEV_1_ in COPD [7]. The precise function and regulation of BDNF in COPD, however, has yet to be elucidated. BDNF, a key mediator of neuronal plasticity in the adult, has been shown to play a crucial role in acute and chronic inflammatory conditions of the airways. BDNF induces neuronal hyperreactivity leading to airway hyperresponsiveness and cough [8,9] and increases the number and function of airway smooth muscle cells [10]. BDNF is stored in large amounts in platelets and is released from platelets during platelet activation, such as in inflammatory conditions or during serum preparation. The majority (> 95%) of circulating BDNF is stored in alpha granules of the platelets, together with Transforming Growth Factor-β_1_ (TGF-β_1_). TGF-β_1_ has been reported to play a major role in the pathogenesis of fibrosis and emphysema in COPD [11,12].

In patients with allergic asthma, serum and platelet concentrations of BDNF are elevated as compared with healthy controls, and correlate with airway obstruction and airway hyperresponsiveness [9]. Although BDNF and TGF-β_1_ are stored in the same platelet granule, serum TGF-β_1_ concentrations are not significantly different from controls in patients with mild to moderate asthma [9]. In contrast, a detailed investigation of the concentrations of BDNF and other platelet-derived mediators in patients with COPD, and their relationship to lung function and disease severity, is currently lacking. It was the aim of the present study, therefore, to investigate serum concentrations of BDNF and other platelet-derived mediators and lung function in patients with different stages of COPD and in non-COPD controls. We hypothesised that the expression of BDNF and TGF-β_1_ and the association with lung function might differ between asthma and COPD.

## Methods

### Study population

All participants were recruited at a German rehabilitation centre from February through September 2011. Sixty-three patients with an already established diagnosis of COPD were recruited. Inclusion criteria were as follows: (1) a history of active or passive tobacco smoking, (2) a ratio of post-bronchodilator forced expiratory volume in one second (FEV_1_) to forced vital capacity (FVC) of < 70 %, and (3) an increase of less than 10% of FEV_1_ following inhalation of 400 μg of salbutamol. Exclusion criteria were: (1) acute exacerbations of COPD or acute infections in the last 4 weeks prior to exclusion, (2) other chronic inflammatory or autoimmune conditions, (3) any malignant disorder and (4) treatment with clopidogrel. The latter exclusion criterium was chosen because clopidogrel treatment, but not aspirin treatment, inhibits the release of BDNF from human platelets and has, therefore, an impact on BDNF serum levels in humans [13]. For the classification of disease severity, spirometric GOLD stages were used [1]. Seventeen patients matched for age and comorbidities with a history of tobacco smoke inhalation but without clinical signs consistent with COPD were enrolled as a control group. Inclusion criteria for controls were (1) a history of active or passive tobacco smoking, (2) a ratio of post-bronchodilator forced expiratory volume in one second (FEV_1_) to forced vital capacity (FVC) of > 70 %. Exclusion criteria for controls were (1) any chronic inflammatory or autoimmune conditions, (2) any malignant disorder and (3) treatment with clopidogrel. All patients and controls provided their written informed consent prior to enrollment. The study was approved by the local Ethics committee of Rostock, Germany.

### Measurements of laboratory parameters and lung function

Peripheral blood was drawn from a cubital vein of the participants. Serum samples were prepared by resting the collected blood in additive-free containers for 60 minutes at room temperature (to ensure complete clotting and platelet degranulation in the sample), followed by centrifugation at 2000 x g for 15 minutes at room temperature. Aliquots of serum samples were stored at – 20° C until measurement. Serum concentrations of BDNF and TGF-β_1_ were measured by ELISA as described [14]. Serum levels of Serotonin (5-Hydroxytriptamine, 5-HT) were measured by High Performance Liquid Chromatography (HPLC) as described [14]. Total and differential blood cell counts were measured using EDTA-containers as described [14]. Lung function parameters were assessed using a body plethysmograph (CareFusion, Hoechberg, Germany) as described [9].

### Statistical analysis

Statistical analysis was performed using SPSS Statistics 17.0 (SPSS Inc., Chicago, Illinois, USA). The majority of parameters were not normally distributed. Therefore, parameters were expressed as medians (minimum – maximum). General differences in BDNF, Serotonin and TGF-β_1_ concentrations between the groups were analysed using the Kruskal-Wallis test. For direct comparisons of specific groups, the Mann–Whitney-U test for unrelated samples was used. For the analysis of trends in BDNF, Serotonin and TGF-β_1_ concentrations, the Jonkheere-Terpstra test was performed. In order to explore possible influencing factors for the observed differences in BDNF and TGF-ß_1_ concentrations in serum, the following parameters, which showed at least a mild correlation (r > 0.3) in an univariate analysis with BDNF or TGF-ß_1_ concentrations in serum, were enrolled into a multivariate analysis: BDNF concentration in serum, TGF-ß_1_ concentration in serum, Serotonin concentration in serum, FEV_1_, platelet concentration in peripheral blood, body mass index (BMI). Correlation analyses were performed using Spearman’s correlation coefficient. Probability values of p<0.05 were regarded as significant.

## Results

### Characteristics of the participants

**Figure 1:**
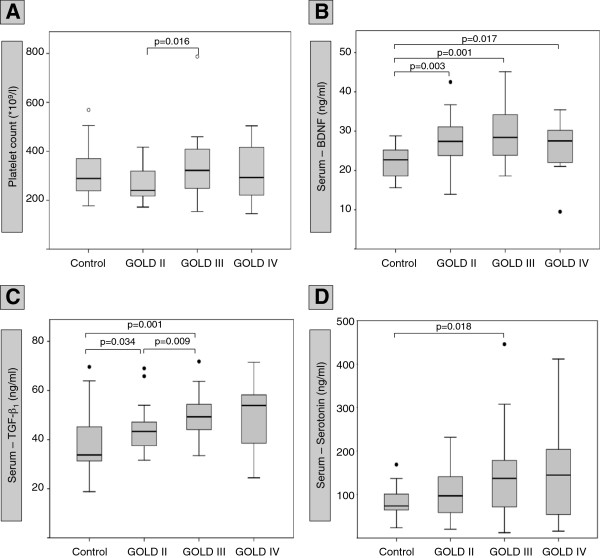
**Platelet counts and serum levels of platelet-derived mediators**. The Figure shows platelet counts (A) and serum levels (ng/ml) of BDNF (B), TGF-β1 (C) and Serotonin (D) in different stages of COPD (GOLD II, GOLD III, GOLD IV) and in controls. The median (line within the box), interquartile range (edges of the box), the range of values that are not outliers (vertical lines) and outliers (cases more distant than 1.5 interquartile ranges from the upper or lower quartile: dots) are shown. Asterisks mark significant differences between the groups. Probability values of p<0.05 were regarded as statistically significant.

The clinical characteristics of the different groups are detailed in Table 1. A total of 63 patients (female: n=24, male: n=39) with COPD (22 patients with spirometric GOLD stage 2, 28 patients with spirometric GOLD stage 3, and 13 patients with spirometric GOLD stage 4), and 17 comorbidity-matched controls (female: n=5, male: n=12) were analysed. Of the 63 COPD patients, 59 (94%) were treated with long-acting β-agonists (LABA), 58 (92%) with long-acting muscarinic antagonists (LAMA) and 49 (78%) with inhaled corticosteroids (ICS). Thirteen patients (21%) were treated with oral corticosteroids (OCS). Five patients (8 % of all participants) were treated with the PDE-4-inhibitor roflumilast. Five patients (8 % of all patients) were taking antidepressants: GOLD stage 2 sertraline (n=1) and opipramole (n=1); GOLD stage 3 citaloprame (n=1); GOLD stage 4 citaloprame (n=1) and doxepine (n=1). None of the comorbidity-matched 17 controls was treated with either of the aforementioned drugs (Tab. 1). There were no significant differences in age or body mass index between the groups (Tab. 1). Patients in all 3 COPD groups had significantly more pack years than controls (Tab. 1). All lung function parameters differed significantly between controls and the 3 groups of patients with COPD (Tab. 1). All stages of COPD presented with significantly elevated hemoglobin and hematocrit levels as compared to non-COPD controls. In contrast, there were no differences in platelet or leukocyte counts between controls and COPD subgroups (Tab. 1). The only significant difference in platelet counts was observed between COPD GOLD 2 (239 x 10^9^ platelets/l) and GOLD 3 (322 x 10^9^ platelets/l, p<0.05) (Figure 1A).

**Table 1:** Participants characteristics

	**COPD**				**Control**	**p**
	all	GOLD 2	GOLD 3	GOLD 4		COPD (all) vs. Control
	n = 63	n = 22	n = 28	n = 13	n = 17	
**Age**	64.0	67.0	63.0	62.0	62.0	0.96
(years)	[43…79]	[45…78]	[43…76]	[51…79]	[51…73]	
**Body Mass Index**	24.8	29.4	23.4	23.8	27.4	0.40
(kg*m^-2^)	[13.6…46.5]	[19.3…39.7]	[13.6…46.5]	[15.8…32.0]	[22.9…57.1]	
**pack years**	40.0	41.5	42.0	34.0	25.0	< 0.001
	[0…100]	[0…100]	[18…75]	[20…60]	[20…40]	
**Smoking history**						-
never/active/former	2 / 11 / 48	1 / 6 / 14	1 / 5 / 21	0 / 0 / 13	0 / 1 / 16	
**Inhaled medication**						-
LABA/LAMA/ICS	59 / 58 / 49	21 / 19 / 20	25 / 26 / 18	13 / 13 / 11	0 / 0 / 0	
**Oral medication**						-
OCS/PDE-I/TPH	13 / 5 / 10	3 / 0 / 2	7 / 2 / 5	3 / 3 / 3	0 / 0 / 0	
**Hemoglobin**	14.4	14.3	14.7	13.8	11.6	< 0.001
(mg/l)	[10.3…16.9]	[10.3…16.4]	[10.4…16.9]	[11.6…16.8]	[9.6…14.8]	
**Hematocrit**	42.4	42.4	42.8	42.0	35.7	< 0.001
(%)	[31.1…49.9]	[31.1…47.4]	[31.1…49.9]	[37.6…49.4]	[27.8…41.6]	
**Platelets**	292	239	322	293	289	0.56
(*10^9^/l)	[145…787]	[172…417]	[154…787]	[145…504]	[177…569]	
**Leukocytes**	8.6	8.1	9.4	8.7	8.7	0.69
(*10^9^/l)	[6.1…18.3]	[6.2…11.9]	[6.1…18.3]	[6.8…17.8]	[6.4…11.6]	
**FEV**_**1**_**total**	1.13	1.46	1.11	0.69	2.32	< 0.001
(l)	[0.45…2.34]	[0.96…2.34]	[0.76…1.76]	[0.45…0.91]	[1.33…4.24]	
**FEV**_**1**_**% pred.**	42.6	58.2	41.1	27.4	76.1	< 0.001
(%)	[16.3…76.2]	[50.3…76.2]	[30.0…48.5]	[16.3…29.7]	[55.2…145.8]	
**FEV**_**1**_**% VC**	46.9	57.8	45.3	34.5	83.3	< 0.001
(%)	[22.2…67.0]	[41.7…67.0]	[30.2…63.8]	[22.2…49.3]	[71.0…99.7]	
**MEF25 total**	0.25	0.31	0.25	0.17	1.02	< 0.001
(l/s)	[0.10…0.62]	[0.13…0.62]	[0.12…0.38]	[0.10…0.36]	[0.36…4.58]	
**MEF25 % pred.**	18.8	24.7	17.5	12.9	62.3	< 0.001
(%)	[7.3…41.4]	[10.9…41.4]	[8.8…32.1]	[7.3…30.0]	[29.6…286.0]	
**RV total**	4.46	3.62	4.76	6.05	2.60	< 0.001
(l)	[2.27…8.48]	[2.72…5.37]	[2.27…8.48]	[3.66…8.26]	[1.12…3.76]	
**RV % pred.**	200.6	158.5	203.6	273.7	113.9	< 0.001
(%)	[101.9…376.8]	[114.0…240.1]	[101.9…376.8]	[204.5…317.5]	[48.1…146.8]	
**TLC total**	7.09	6.17	7.21	8.46	5.40	< 0.01
(l)	[4.1…14.09]	[5.1…8.9]	[4.1…11.7]	[5.0.…14.1]	[3.0…8.2]	
**TLC % pred.**	116.8	111.1	116.8	135.8	84.5	< 0.001
(%)	[71.5…179.2]	[83.3…149.1]	[71.5…169.5]	[107.5…179.2]	[52.3…111.0]	
**RV % TLC**	64.8	57.4	65.2	73.5	50.1	< 0.001
(%)	[45.1…86.2]	[45.1…70.6]	[52.3…73.6]	[65.4…86.2]	[26.5…63.5]	
**TLCO / VA total**	0.69	0.84	0.64	0.37	1.03	< 0.001
(mmol/min/kPa/l)	[0.01…1.72]	[0.37…1.72]	[0.13…1.27]	[0.01…0.90]	[0.72…1.91]	
**TLCO / VA % pred.**	49.0	60.3	42.3	27.8	77.6	< 0.001
(%)	[0.95…105.4]	[25.6…105.4]	[8.0…89.1]	[0.95…68.6]	[57.3…138.5]	

Data are shown as median [minimum…maximum]. LABA – long-acting beta agonist, LAMA – long acting muscarinic antagonist, ICD – inhaled corticosteroids, OCS – oral corticosteroids, PDE-I – phosphodiesterase inbibitor, TPH – theophylline.

### Serum concentrations of BDNF and other platelet-derived mediators

The general analysis of serum mediators using the Kruskal-Wallis test revealed that there were statistically significant differences in BDNF and TGF-β_1_ serum concentrations, but not Serotonin serum concentrations, between controls and patients with COPD. Direct comparisons between the groups showed that serum concentrations of BDNF were significantly elevated in all COPD subgroups compared to non-COPD controls (Figure 1B). In contrast, there were no significant differences in serum BDNF concentrations between COPD subgroups, although there were highest median serum BDNF concentrations in spirometric GOLD stage 3 (Figure 1B). In order to relate the observed changes of BDNF to other platelet-derived mediators, direct comparisons of the subgroups were performed for TGF-β_1_ (which is stored in platelet alpha granules) and Serotonin (5-Hydroxytryptamine, 5-HT, which is stored in platelet dense-core granules). In patients with spirometric GOLD stages 2 and 3, TGF-β_1_ serum levels were significantly elevated compared to non-COPD controls (Figure 1C). There were highest median TGF-β_1_ serum levels (median: 53.9 ng/ml) in patients with spirometric GOLD stage 4, but this was not significant (Figure 1C). Due to the high variability of 5-HT serum concentrations in patients with COPD, the only statistically significant difference was found between controls and patients with spirometric GOLD stage 3 (Figure 1D). The Jonkheere-Terpstra test revealed that there was a trend to increasing BDNF and TGF-β_1_ serum concentrations (but not Serotonin serum concentrations) with increasing COPD severity.

**Figure 2:**
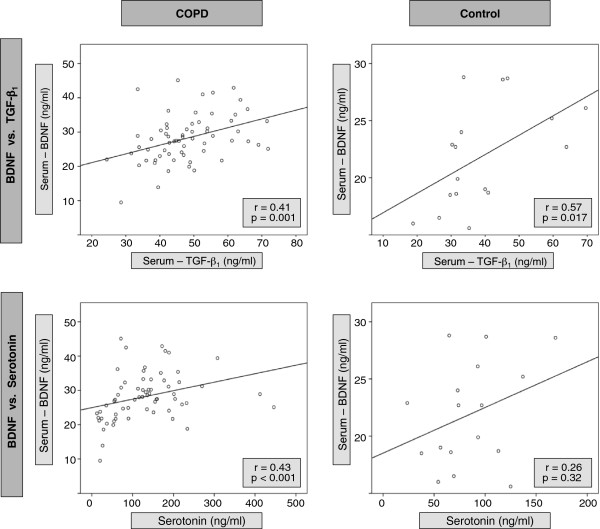
**Correlation of platelet-derived mediators BDNF, TGF-β1 and Serotonin in COPD patients and controls**. Shown in the upper line are correlations between serum levels (ng/ml) of BDNF and TGF-β1 for all COPD patients and controls. The lower line shows correlations between serum levels (ng/ml) of BDNF and Serotonin for all COPD patients and controls. Each dot represents one patient; the line is the regression line calculated with SPSS (Chicago, Illinois, USA). Spearman’s rank correlation coefficient (r) and the significance of the correlation (p) are given in the graph.

Serum concentrations of BDNF correlated significantly with serum concentrations of TGF-β_1_, both in patients with COPD and in controls (Figure 2). In contrast, BDNF serum concentrations correlated with 5-HT serum concentrations only in patients with COPD, but not in controls (Figure 2). There were no significant differences in serum levels of BDNF, TGF-β_1_ and Serotonin between patients treated with ICS (n=49) or OCS (n=13) and those not treated with ICS (n=14) or OCS (n=50). In addition, there were no significant differences in BDNF, TGF-β_1_ and Serotonin serum levels between patients treated with antidepressants (n=5) or roflumilast (n=5) and those not treated with antidepressants (n=58) or roflumilast (n=58). The multivariate analysis revealed that only TGF-ß_1_ in serum and Serotonin in serum were significantly associated with serum BDNF concentrations, and only BDNF in serum and platelet counts were significantly associated with serum TGF-ß_1_ concentrations.

### Correlation of platelet-derived mediators with lung function

**Figure 3:**
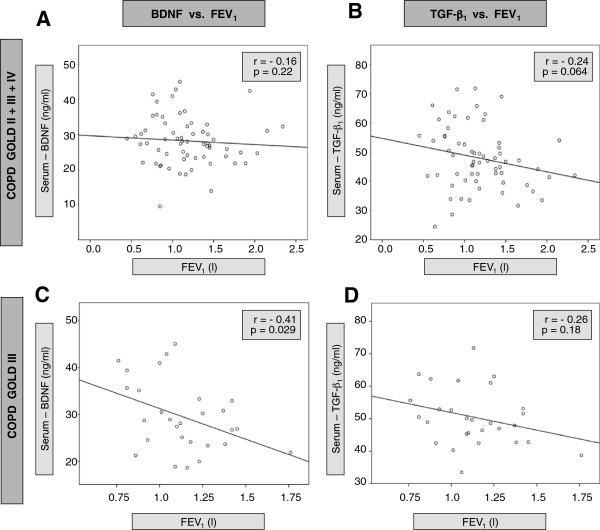
**Correlation of serum BDNF and TGF-β1 with FEV1 (forced exspiratory volume in 1 second) in patients with COPD**. Shown in the left column are correlations between serum levels of BDNF (ng/ml) and FEV1 for all COPD patients (A) and patients with COPD stage GOLD III (C). The right column shows correlations between serum levels of TGF-β1 (ng/ml) and FEV1 for all COPD patients (B) and patients with COPD stage GOLD III (D). Each dot represents one patient; the line is the regression line calculated with SPSS (Chicago, Illinois, USA). Spearman’s rank correlation coefficient (r) and the significance of the correlation (p) are given in the graph.

**Figure 4:**
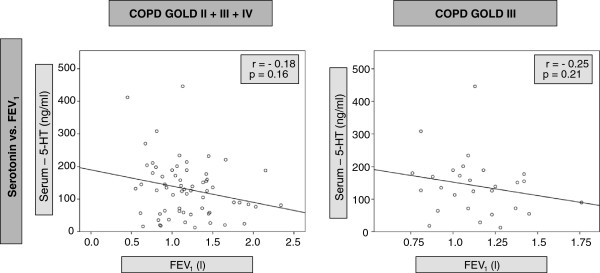
**Correlation of Serotonin (5-HT) with FEV1 (forced exspiratory volume in 1 second) in COPD patients**. Shown are correlations between serum levels of Serotonin (ng/ml) and FEV1 for all COPD patients and subjects with COPD stage GOLD III. Each dot represents one patient; the line is the regression line calculated with SPSS (Chicago, Illinois, USA). Spearman’s rank correlation coefficient (r) and the significance of the correlation (p) are given in the graph.

In the total group of patients with COPD, there was no significant correlation between serum BDNF and FEV_1_ (% predicted), but a trend to a negative correlation between serum TGF-β_1_ and FEV_1_ (% predicted) (Figure 3). In contrast, subgroup analysis revealed a negative correlation between FEV_1_ and serum BDNF (r=−0.41, p<0.05), but not serum TGF-β_1_, in patients with spirometric GOLD stage 3 (the largest subgroup of patients). In contrast, there were no correlations between FEV_1_ and serum concentrations of 5-HT, neither in the group of all COPD patients nor in one of the subgroups (Figure 4). In addition, there were no significant correlations between TGF-β_1_ and emphysema markers (residual volume and carbon monoxide diffusion capacity), neither in the total group of patients nor in the COPD subgroups (data not shown).

## Discussion

There is growing evidence suggesting a crucial role of neurotrophins such as BDNF in the pathogenesis of obstructive airway diseases [7,9,15]. In patients with allergic asthma, a close relationship between elevated systemic BDNF concentrations and lung function has been demonstrated, whereas systemic TGF-β_1_ concentrations were normal [9,16]. This clinical study is the first to show that, in contrast to asthma, COPD is characterised by an elevation of both BDNF and TGF-β_1_ in serum. In addition, we found a stage-dependent association of these platelet mediators with lung function in COPD.

BDNF and TGF-β_1_ are both stored in the alpha-granules of human platelets and are released by degranulation after activation [17]. The neurotrophin BDNF is a key mediator of neuronal and smooth muscle plasticity [8-10], whereas TGF-β_1_ plays a major role in the pathogenesis of connective tissue remodeling and fibrosis [11,12]. Altered platelet activity has been shown both in patients with COPD [18] and in patients with asthma [19], however, the regulation of platelet-derived mediators in obstructive airway diseases is still incompletely understood. It was previously shown that serum and platelet concentrations of BDNF (but not TGF-β_1_) are elevated in patients with mild to moderate allergic asthma, correlating with airway obstruction and airway hyperresponsiveness [9]. These clinical data confirmed findings from animal models of allergic asthma, which showed a crucial role of BDNF in the pathogenesis of airway hyperresponsiveness and airway obstruction [20,21]. Altered BDNF regulation may also contribute to adverse effects of β_2_-agonists in asthma [22]. Salmeterol monotherapy significantly increased systemic BDNF concentrations in patients with asthma, and these changes correlated with the deterioration of airway hyperresponsiveness [16]. Increased BDNF concentrations and increased airway hyperresponsiveness were both reduced after concomitant therapy with fluticasone [16] confirming previous data showing a suppression of BDNF production by corticosteroids [9,23].

In the present study, a significant increase in BDNF serum concentrations was also found in patients with COPD. This finding is in line with experimental data showing systemic overproduction of BDNF in a rat model of COPD [24]. A recent study examining serum concentrations of a broad panel of 92 inflammation-associated analytes postulated that BDNF is among the three most highly elevated mediators in COPD (CD40 Ligand, Epithelial Growth Factor, BDNF) [6]. The analysis of possible confounding factors for the increase in BDNF concentrations in patients with COPD in our study showed that (in addition to the diagnosis of COPD) only platelet mediators significantly influenced BDNF concentrations in serum. This finding confirms the concept that BDNF in serum is derived from platelets. Of note, there were no significant differences in platelet concentrations between controls and patients with COPD, indicating that elevated BDNF concentrations in patients with COPD are not explained by numeric differences in platelets in peripheral blood. Instead, increased concentrations of BDNF in platelet granules in patients with COPD appear to be the most likely explanation for the observed elevation of serum BDNF in COPD. However, platelet concentrations were significantly higher in GOLD stage 3 as compared to GOLD stage 2. Therefore, it cannot be excluded that the trend to an increase of BDNF concentrations in GOLD stage 3 (as compared to GOLD stage 2) might be explained by an elevation of platelet concentrations in peripheral blood.

The large majority of patients (94%), but none of the controls examined in our study were treated with LABAs. Thus, since therapy with the LABA salmeterol was shown to increase BDNF concentrations in serum of patients with asthma [16], it cannot be excluded that the difference between patients and controls is attributable to LABA therapy. However, several lines of evidence suggest that LABA therapy is not the sole explanation for elevated BDNF serum concentrations in patients with COPD. First, a previous study in animals not treated with LABAs showed a systemic increase of BDNF in a model of COPD [24]. Secondly, in contrast to patients with asthma [16], neither inhaled nor oral corticosteroids had an impact on BDNF serum levels in patients with COPD, suggesting that patients with COPD and asthma might differ in their BDNF response to treatment. Finally, LABA monotherapy is a safe first-line therapeutic option in patients with COPD, whereas LABA monotherapy is harmful in patients with asthma [16]. Thus, the lack of detrimental effects of LABAs on COPD control suggests that LABAs do not have the same effect on systemic BDNF concentrations in patients with COPD as in patients with asthma. Taken together, we speculate that enhanced BDNF serum levels observed in patients with COPD were not attributable to pharmacological treatment, but represent a genuine characteristic of this disease.

There is growing evidence from animal models that antidepressants [25] and inhibitors of the phosphopdiesterase 4 (PDE-4) [26,27] can increase BDNF production. In addition, corticosteroids have been postulated to decrease BDNF production [9,23]. In contrast to the controls, a small fraction of patients with COPD was treated with antidepressants (8%), roflumilast (8%) or oral corticosteroids (21%). Although we did not find significant differences in BDNF concentrations between patients treated with antidepressants, roflumilast or oral corticosteroids and those patients not treated with these drugs, our study was clearly underpowered to draw any firm conclusions on the influence of different classes of antidepressants, roflumilast or oral corticosteroids on BDNF serum concentrations in patients with COPD. Further studies with higher percentages of patients with COPD treated with antidepressants, roflumilast or oral corticosteroids are needed to explore this issue.

In contrast to patients with asthma, highest BDNF concentrations were not found in patients with most severe disease. Instead, highest BDNF concentrations were found in patients with spirometric GOLD stage 3. Of note, in this subgroup of patients, a significant negative correlation between FEV_1_ and serum BDNF was found, which was comparable to the findings in asthma [9]. The lacking association of serum BDNF with FEV_1_ in the total group of patients with COPD could have 3 reasons: (1) parameters of airflow limitation could be confounded by emphysema development, (2) the maximum of BDNF production and BDNF effects could take place in patients with less severe disease, and (3) BDNF serum concentrations could be confounded by a concomitant depressive disorder of the patients. Firstly, the reduction in FEV_1_ in COPD is both due to airflow limitation and due to the presence of emphysema. All patients with spirometric GOLD stage 4 analysed in our study had a residual volume (RV) > 200 % of the predicted value, suggesting that severe emphysema was a major determinant of the observed FEV_1_ reduction in this subgroup. In contrast, the RV was significantly lower in the subgroup of patients with spirometric GOLD stage 3, which implies that the relative contribution of airflow limitation to the FEV_1_ reduction was higher in the group with spirometric GOLD stage 3 than in the group with spirometric GOLD stage 4. Thus, it might be speculated that the disappearance of the association between BDNF and FEV_1_ in spirometric GOLD stage 4 was due to a strong influence of emphysema on the FEV_1_ reduction in this subgroup. Secondly, it is conceivable that the BDNF production and the subsequent BDNF effects on airflow limitation are most pronounced in patients with spirometric GOLD stage 3. It has been shown that mononuclear cells (such as lymphocytes and monocytes) are important sources of BDNF in inflammatory conditions [9]. Of note, the number of lymphoid follicles in airway walls (which are important markers of small-airway inflammation in COPD) peaks in spirometric GOLD stage 3 [28]. Thus, it is conceivable that BDNF might play a major role in lung function reduction in COPD in those patients with most severe small-airway inflammation (which are not necessarily those patients with most severe FEV_1_ reduction). Finally, depression, which is common in patients with severe COPD [29], could reduce BDNF serum concentrations in patients with severe COPD. There is strong evidence from the literature indicating that depression reduces serum BDNF concentrations in humans [25]. Thus, concomitant depression could mask a possible inflammatory BDNF overproduction in patients with severe COPD. This study was not designed to investigate the association of BDNF levels and the prevalence of depressive disorders in specific stages of COPD. Therefore, future studies focusing on this particular issue are warranted.

In contrast to patients with allergic asthma, there was an increase in TGF-β_1_ concentrations in patients with COPD. In addition, there was a correlation of BDNF with TGF-β_1_ in these patients, which was not found in patients with asthma [9]. Thus, it appears that, in contrast to asthma, COPD is characterised by a concomitant increase of both platelet-mediators BDNF and TGF-β_1_. However, there were also stage-dependent differences between the mediators: median serum BDNF concentrations peaked in patients with spirometric GOLD stage 3, whereas highest median concentrations of TGF-β_1_ were found in patients with spirometric GOLD stage 4. TGF-β_1_ has a multitude of effects in the organism. One of them is an immunosuppressive effect on humoral (such as inhibition of interleukin-2 production) and cellular (such as inhibition of proliferation and differentiation of T-cells) components of the immune system [11]. In addition to the anti-inflammatory effect, TGF-β_1_ is a potent inductor of airway fibrosis and extracellular deposition of collagen [30]. In patients with COPD, increased expression of TGF-β_1_ in the airway epithelium has been associated with enhanced fibrotic airway remodeling [31]. Other reports postulated that increased TGF-β_1_ expression in COPD is predominantly vessel-associated [32]. In addition to TGF-β_1_ overproduction, altered TGF-β_1_ signaling and several TGF-β_1_ polymorphisms have been postulated to play a role in the pathogenesis of COPD and emphysema [33,34]. Our observation that median TGF-β_1_ serum concentrations were rising with increasing disease severity supports the postulate that this mediator plays a role in airway remodeling in COPD. However, we did not find a significant association of TGF-β_1_ serum concentrations with markers of emphysema (residual volume and the carbon monoxide diffusion capacity), suggesting that the role of TGF-β_1_ in the pathogenesis of structural changes in COPD might be more complex.

In our study, a trend to elevated serum concentrations of Serotonin (5-HT) was found in patients with COPD as compared to controls. In patients with COPD in spirometric GOLD stage 3, this elevation was statistically significant. In a recently published study, plasma concentrations of serotonin (representing circulating serotonin and not serotonin released from platelets after serum preparation) were shown to be elevated in patients with stable COPD [35]. In both studies (the previous study with plasma serotonin and our study with serum serotonin), there was no significant association of serotonin concentrations and lung function. Thus, it might be hypothesized that there is an elevation of circulating serotonin in stable COPD, but no elevation of serotonin stored in platelets. In addition, the lacking association with lung function might indicate that systemic elevation of serotonin is a general phenomenon in COPD, independent of disease severity. The source of circulating serotonin in COPD, however, remains to be elucidated [35].

There is an ongoing debate on similarities and differences between asthma and COPD [36]. The so-called Dutch hypothesis postulates that both diseases have common genetic characteristics, which are only modified by different environmental stimuli, leading to a more asthma-like or a more COPD-like phenotype [37]. Other authors argue against this hypothesis, by stressing the substantial differences in clinical presentation, pathology and response to therapy between asthma and COPD [38]. The results of the present study reveal both similiarities and differences between the diseases. On one hand, both diseases were characterised by a systemic upregulation of BDNF, a marker of neuromuscular dysfunction of the airways. On the other hand, only COPD was associated with a systemic upregulation of TGF-β_1_, a potent inductor of airway fibrosis and extracellular collagen deposition. Thus, our study supports the idea that there are both similarities and differences between the diseases, which can be disentangled on a molecular level.

## Conclusions

We demonstrate for the first time that, in contrast to asthma, COPD is characterised by increased concentrations of both BDNF and TGF-β_1_ in serum. The stage-dependent association with lung function supports the hypothesis that these platelet mediators may play a role in the pathogenesis of COPD.

## Competing interests

The authors declare that they have no competing interest.

## Authors’ contribution

PS, JCV, ML: designed the study, analysed and interpreted the data. UW, KB, CZ participated in patient acquisition, collection of samples and measurements. PS, UW, KB, CZ, JCV, ML made important intellectual contribution to the draft and revision of the manuscript. PS, UW, KB, CZ, JCV, ML approved the final version of the manuscript.
